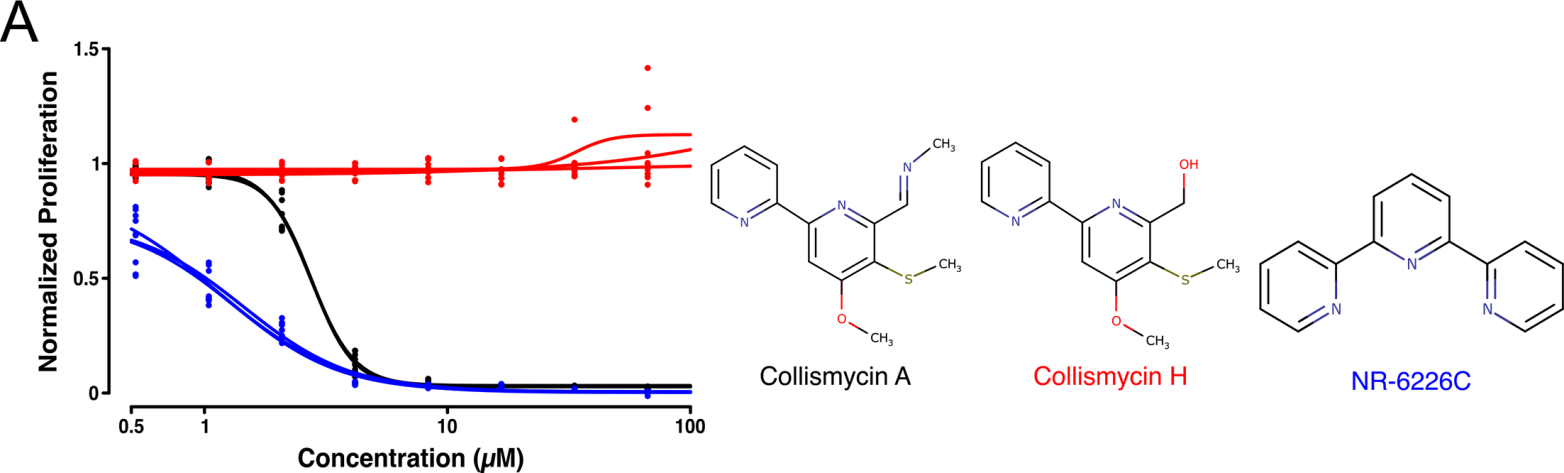# Correction for Corrales et al., “Characterization of a selective, iron-chelating antifungal compound that disrupts fungal metabolism and synergizes with fluconazole”

**DOI:** 10.1128/spectrum.00730-25

**Published:** 2025-10-21

**Authors:** Jeanne Corrales, Lucia Ramos-Alonso, Javier González-Sabín, Nicolás Ríos-Lombardía, Nuria Trevijano-Contador, Henriette Engen Berg, Frøydis Sved Skottvoll, Francisco Moris, Oscar Zaragoza, Pierre Chymkowitch, Ignacio Garcia, Jorrit M. Enserink

## AUTHOR CORRECTION

Volume 12, no. 2, e02594-23, 2024, https://journals.asm.org/doi/10.1128/spectrum.02594-23. Figures 1C and 2A and Fig. S1 should appear as shown in this correction. Regretfully, two nitrogen atoms were positioned incorrectly in some of the chemical structures. We apologize for this error, which does not change the interpretation of the data or the conclusions of the study.

**Fig 1:**
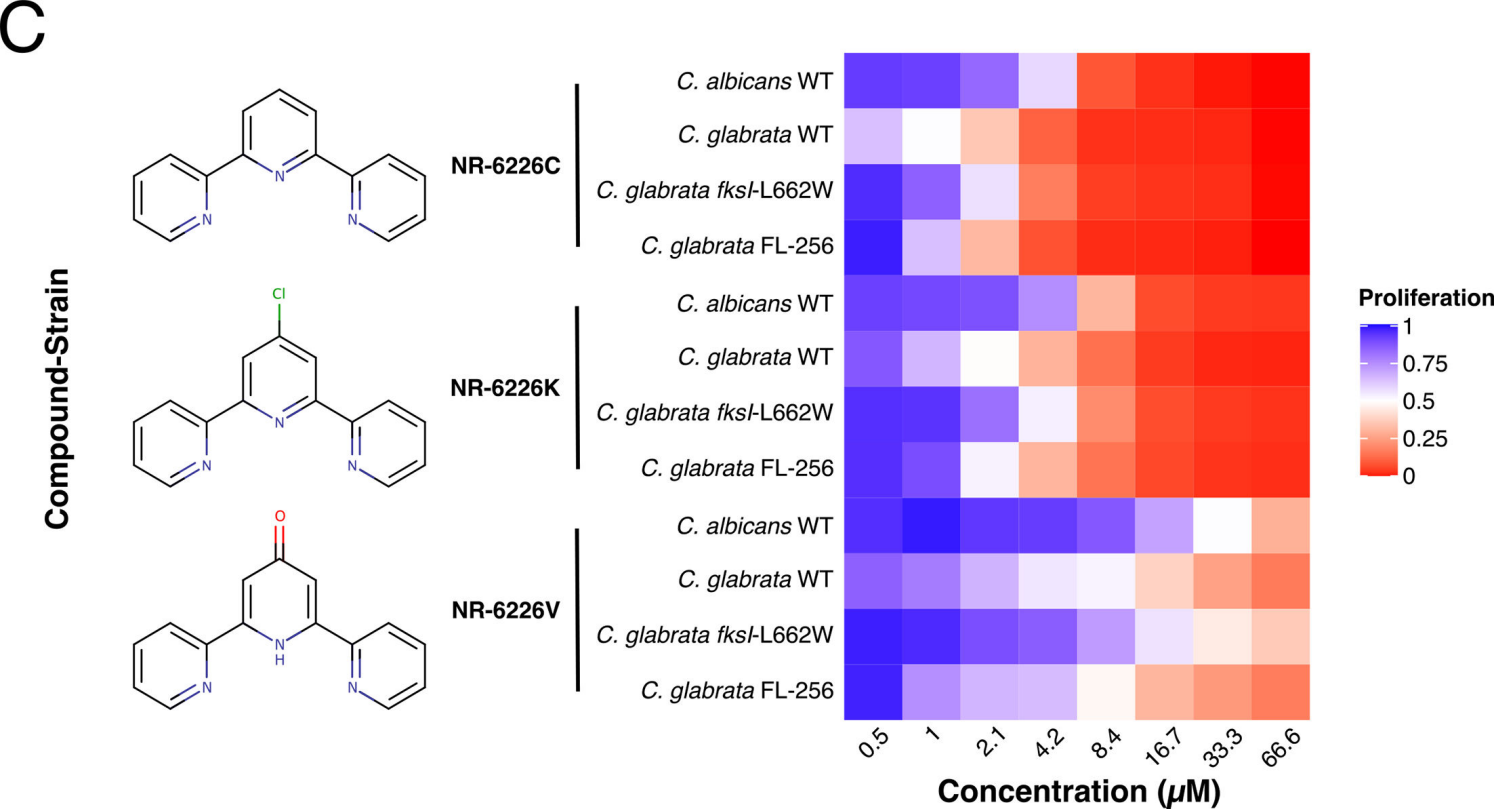


**Fig 2:**