# Supplementary material for: Characterization of a selective, iron-chelating antifungal compound that disrupts fungal metabolism and synergizes with fluconazole
Source: Microbiol Spectr. 2024 Jan 17;12(2):e02594-23. doi: 10.1128/spectrum.02594-23 (PMC10845951; doi:10.1128/spectrum.02594-23)
Supplement: Fig. S1 — Supporting figure. [file spectrum.02594-23-s0001.pdf]

Supplemental Figure 1

Compound	Structure	Compound	Structure
Collismycin 21	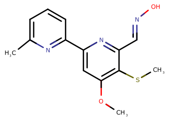	NR-5012	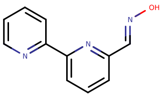
Collismycin 22	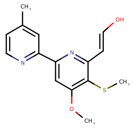	NR-6226A	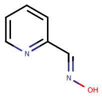
Collismycin 22-ACID	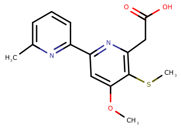	NR-6226B	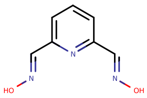
Collismycin A	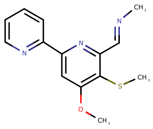	NR-6226C	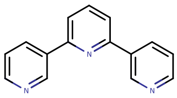
Collismycin DC	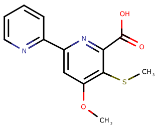	NR-6226D	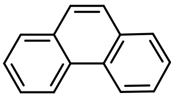
Collismycin DH	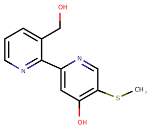	NR-6226K	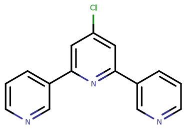
Collismycin H	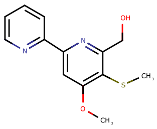	NR-6226V	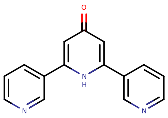
Collismycin H-BUT	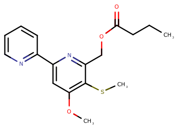	NR-6266A	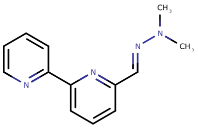
Collismycin HA	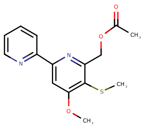	NR-6266B	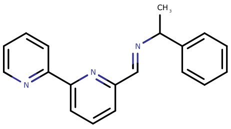
Collismycin SN	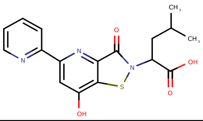	NR-6265P	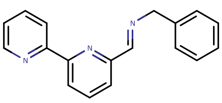
Collismycin SC	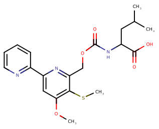	NR-6268A	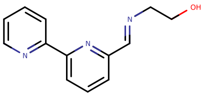
NR-4492C	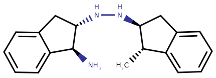	NR-6269A	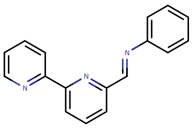
NR-4493C	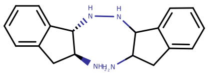	NR-6269B	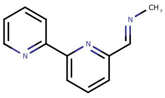

Supplemental Figure S1. Collismycin-related compound names and structures.
