# Supplementary material for: Characterization of a selective, iron-chelating antifungal compound that disrupts fungal metabolism and synergizes with fluconazole
Source: Microbiol Spectr. 2024 Jan 17;12(2):e02594-23. doi: 10.1128/spectrum.02594-23 (PMC10845951; doi:10.1128/spectrum.02594-23)
Supplement: Fig. S2 — Supporting figure. [file spectrum.02594-23-s0002.pdf]

Supplemental Figure 2

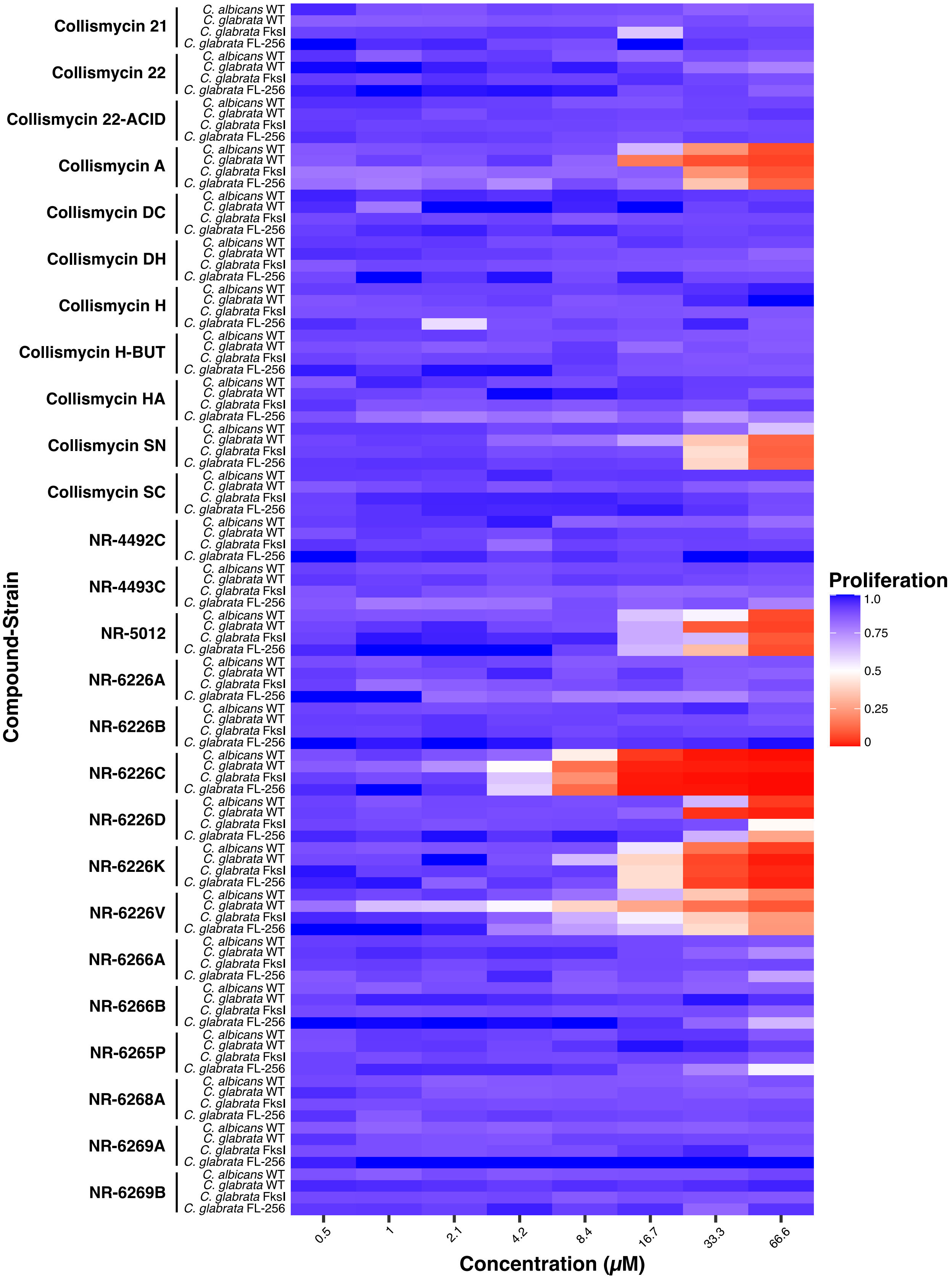

Supplemental Figure S2. Compound screening. Heatmap of *Candida* proliferation after treatment for 24 hrs with the indicated concentrations of Collismycin analogs. Cell growth was measured using OD_{600}. Data were normalized to cells treated with DMSO.
