# Supplementary material for: Characterization of a selective, iron-chelating antifungal compound that disrupts fungal metabolism and synergizes with fluconazole
Source: Microbiol Spectr. 2024 Jan 17;12(2):e02594-23. doi: 10.1128/spectrum.02594-23 (PMC10845951; doi:10.1128/spectrum.02594-23)
Supplement: Fig. S3 — Supporting figure. [file spectrum.02594-23-s0003.pdf]

Supplemental Figure 3

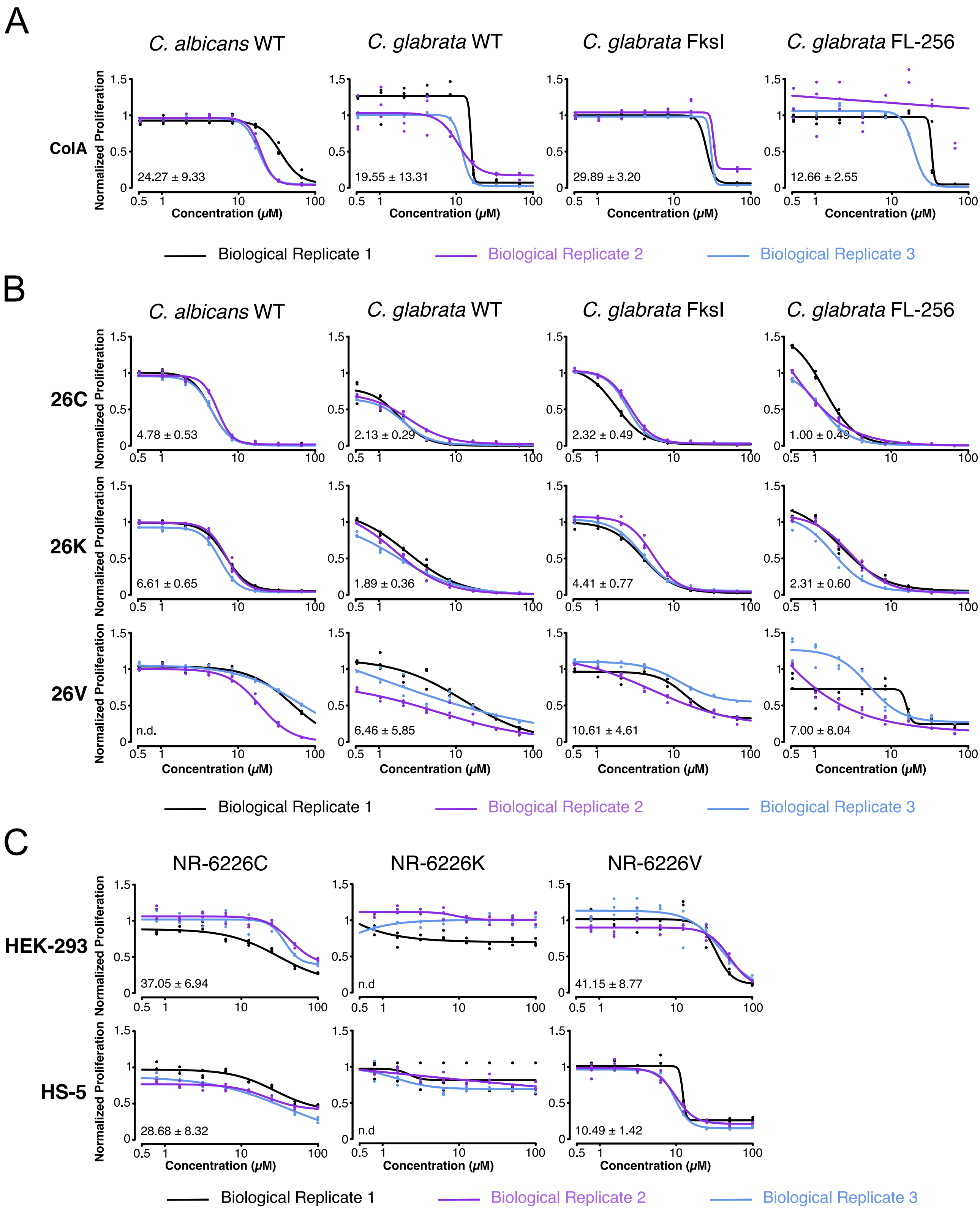

Supplemental Figure S3. ***A, B,*** Proliferation curves and EC_{50} values of *Candida spp* treated for 24 hrs with either Collismycin A (A) or with 26C, 26K, 26V (B). Cell growth was measured using OD_{600}. ***C,*** Proliferation curves and EC_{50} values of HEK-293 and HS-5 cells after treatment for 24 hrs with either 26C, 26K, or 26V. Closed circles indicate technical replicates, lines indicate biological replicates. EC_{50} values were calculated as described in Figure 2C.
