# Supplementary material for: Characterization of a selective, iron-chelating antifungal compound that disrupts fungal metabolism and synergizes with fluconazole
Source: Microbiol Spectr. 2024 Jan 17;12(2):e02594-23. doi: 10.1128/spectrum.02594-23 (PMC10845951; doi:10.1128/spectrum.02594-23)
Supplement: Fig. S4 — Supporting figure. [file spectrum.02594-23-s0004.pdf]

Supplemental Figure 4

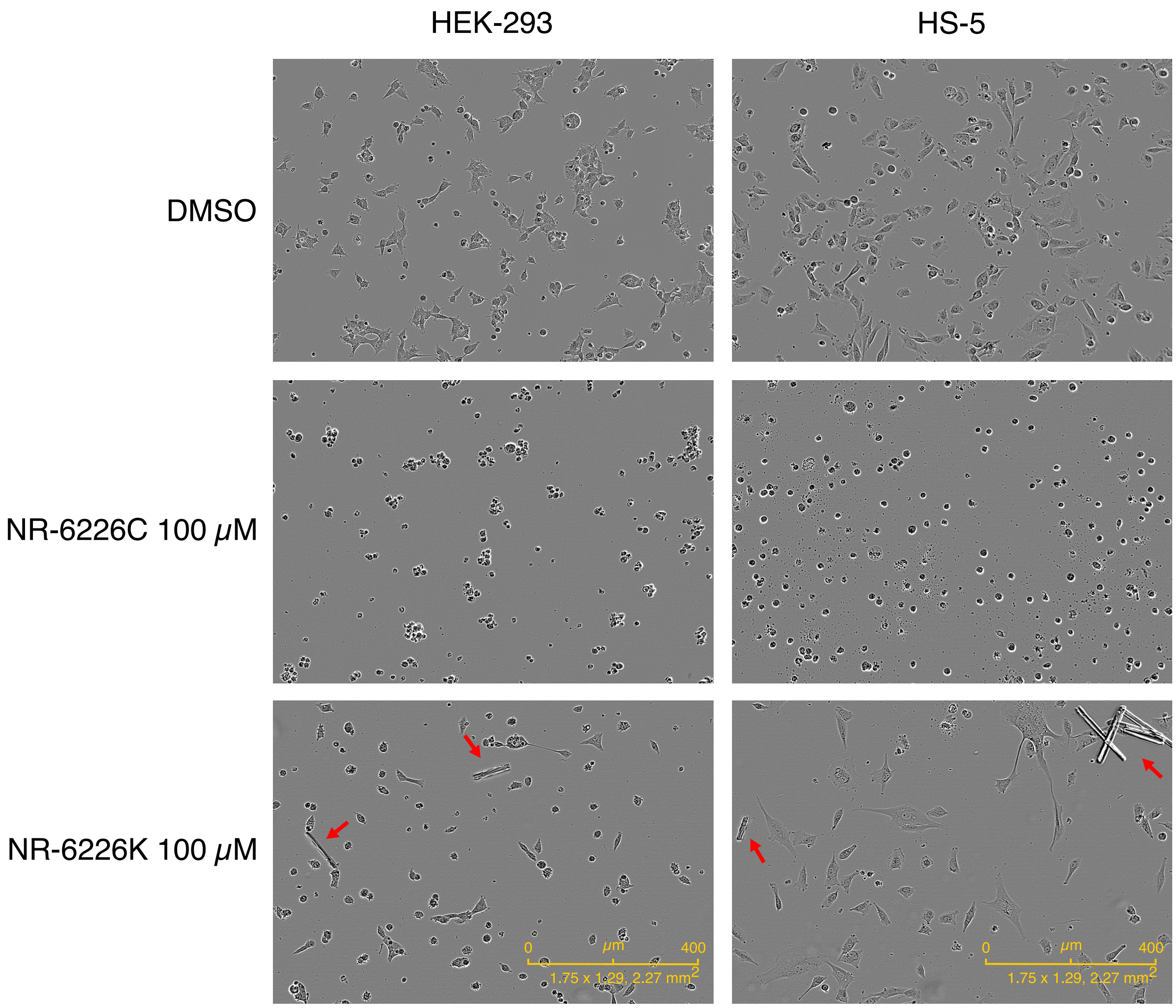

Supplemental Figure S4. HEK-293 and HS-5 cells were treated with either DMSO, 100 μM 26C, or 100 μM 26K and imaged using the Incucyte Live-Cell Analysis System. Red arrows indicate compound precipitation.
