# Supplementary material for: Characterization of a selective, iron-chelating antifungal compound that disrupts fungal metabolism and synergizes with fluconazole
Source: Microbiol Spectr. 2024 Jan 17;12(2):e02594-23. doi: 10.1128/spectrum.02594-23 (PMC10845951; doi:10.1128/spectrum.02594-23)
Supplement: Fig. S5 — Supporting figure. [file spectrum.02594-23-s0005.pdf]

Supplemental Figure 5

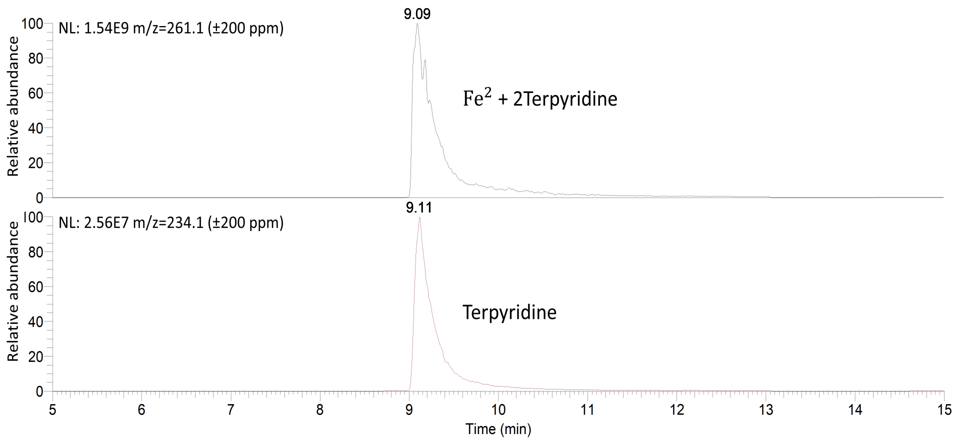

Supplemental Figure S5. Mass spectroscopy of 26C on its own and or bound to Fe^{2+}.
