# Supplementary material for: Characterization of a selective, iron-chelating antifungal compound that disrupts fungal metabolism and synergizes with fluconazole
Source: Microbiol Spectr. 2024 Jan 17;12(2):e02594-23. doi: 10.1128/spectrum.02594-23 (PMC10845951; doi:10.1128/spectrum.02594-23)
Supplement: Fig S6 — Supporting figure. [file spectrum.02594-23-s0006.pdf]

Supplemental Figure 6

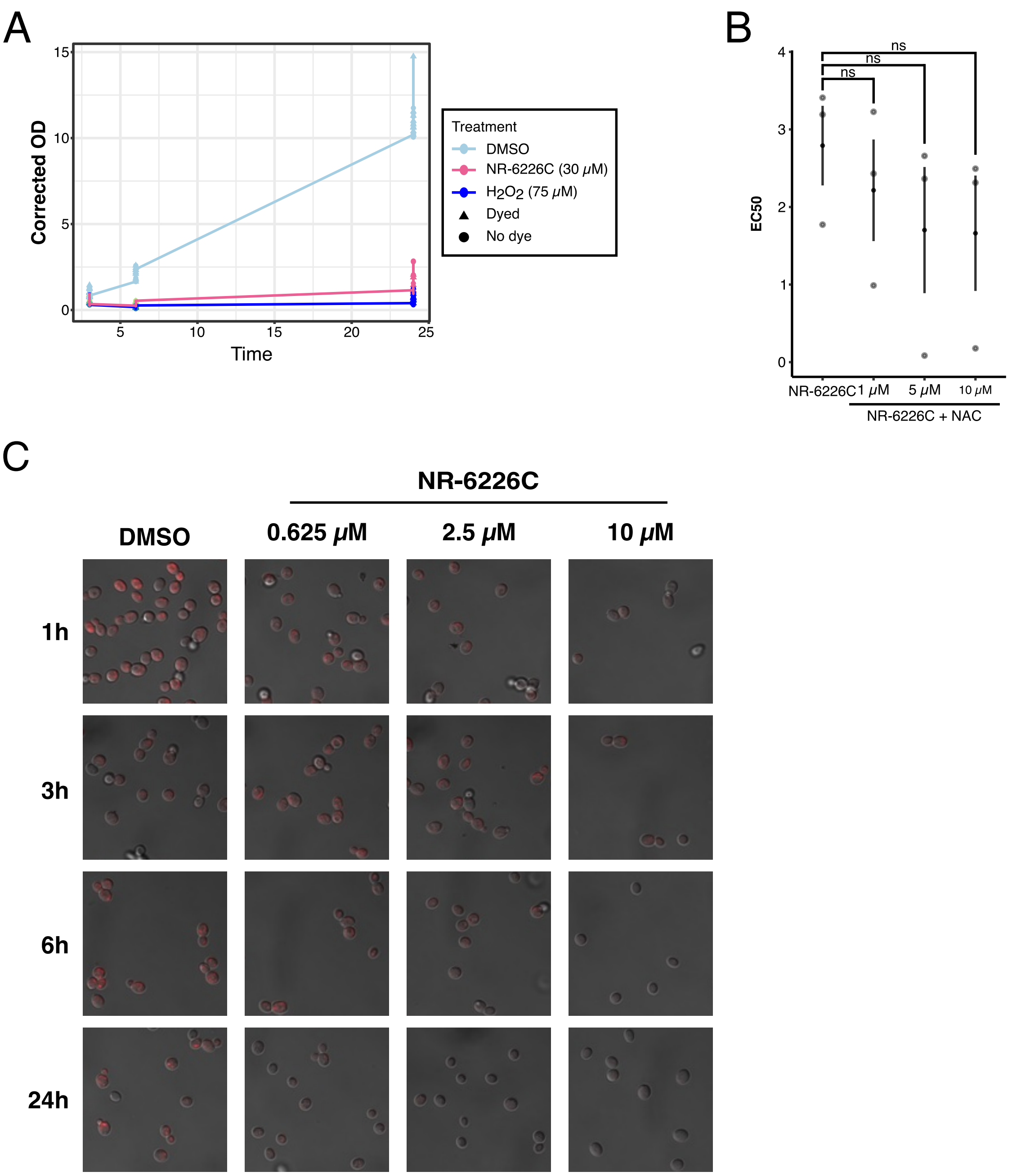

Supplemental Figure S6. ***A,*** Growth curves of *C. glabrata* cells pre-loaded with the H_2DCFDA and then treated with 0.05% DMSO, 30 μM NR-6226C, or 75 μM H_2O_2. Cell proliferation was measured and corrected using OD_{600}. ***B,*** EC_{50} values of *C. glabrata* cells treated with 26C alone within a concentration range of 0.78 μM-100 μM, or with NR-6226C in combination with 1 μM, 5 μM or 10 μM N-Acetyl-L-Cysteine (NAC). Samples were normalized to DMSO controls, followed by EC_{50} estimation. Statistical significance was calculated using one-way ANOVA and Tukey's HSD for post hoc analysis. ***C,*** *C. glabrata* cells were treated with DMSO, 0.625 μM, 2.5 μM or 10 μM NR-6226C for 6 and 24 hrs, after which they were loaded with MitoTracker and imaged using a Zeiss LSM 710 microscope. Representative images of random microscopic fields are shown. ***D,*** *C. glabrata* cells were treated with 0.05 % DMSO, 0.625 μM, 2.5 μM, or 10 μM NR-6226C for 24 hours, then measured for OD_{600nm}. *Statistical* analysis was performed using one-way ANOVA and Tukey's HSD for post hoc analysis. ****P≤0.001; **P≤0.01; ns, not significant; n=3.*
