# Supplementary material for: Characterization of a selective, iron-chelating antifungal compound that disrupts fungal metabolism and synergizes with fluconazole
Source: Microbiol Spectr. 2024 Jan 17;12(2):e02594-23. doi: 10.1128/spectrum.02594-23 (PMC10845951; doi:10.1128/spectrum.02594-23)
Supplement: Fig. S7 — Supporting figure. [file spectrum.02594-23-s0007.pdf]

Supplemental Figure 7

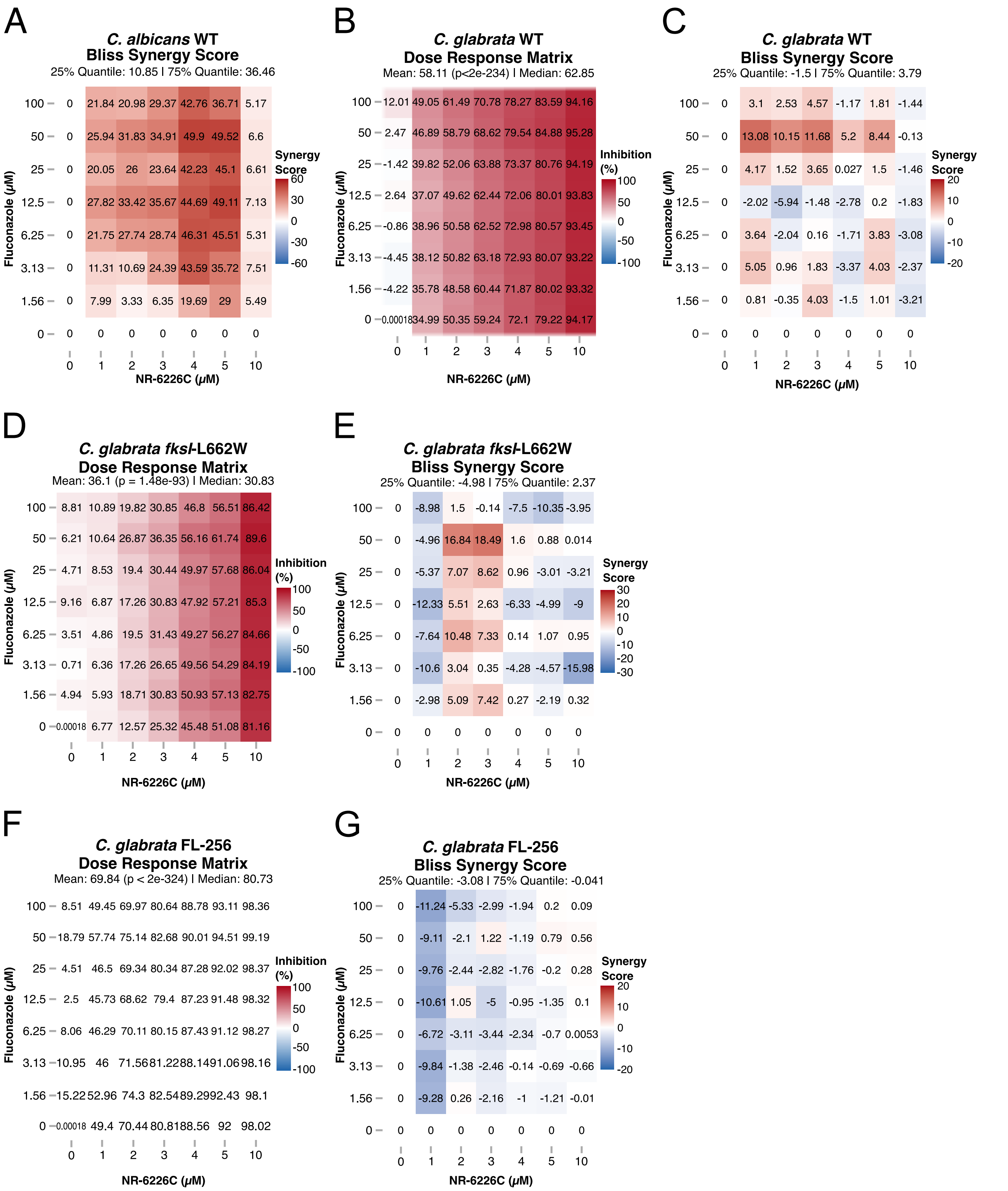

Supplemental Figure S8. ***A, C, E, G,*** Bliss synergy scores of *Candida spp.* treatment with Fluconazole and NR-6226C combination. ***B, D, F,*** Dose response matrix of *Candida glabrata* cells treated in combination with Fluconazole and NR-6226C. Relative cell numbers were quantified as described in Figure 1B. Dose response matrices and synergy scores were obtained using SynergyFinder in R.
