# Supplementary material for: Characterization of a selective, iron-chelating antifungal compound that disrupts fungal metabolism and synergizes with fluconazole
Source: Microbiol Spectr. 2024 Jan 17;12(2):e02594-23. doi: 10.1128/spectrum.02594-23 (PMC10845951; doi:10.1128/spectrum.02594-23)
Supplement: Table S1 — Strain list. [file spectrum.02594-23-s0008.docx]

**Supplemental Table S1.** Yeast strains.

| **Species** | **Strain number** | **Relevant characteristics or genotype** | **Source** |
| --- | --- | --- | --- |
| *C. albicans* | JEY13033 | N/A | CCUG32723 |
| *C. glabrata* | JEY10028 | N/A | ATCC15545 |
| *C. glabrata* | JEY12725 | *fksI*-L662W; Patient-derived from Oslo University Hospital | This study |
| *C. glabrata* | JEY12726 | FL-256; Patient-derived from Oslo University Hospital | This study |
| *C. glabrata* | JEY12527 | 2001HT; *his3Δ trp1Δ* | Kitada, et al. (1995)^53^ |
| *S. cerevisiae* | BY4741 | *MATa his3Δ1, leu2Δ0, met15Δ0, ura3Δ0* | Brachmann, et al. (1998)^54^ |
| *S. cerevisiae* | *aft1*Δ | *MATa his3Δ1, leu2Δ0, met15Δ0, ura3Δ0, aft1::KanMX* | Giaever, et al. (2002)^55^ |
