# Supplementary material for: Correction for Corrales et al., “Characterization of a selective, iron-chelating antifungal compound that disrupts fungal metabolism and synergizes with fluconazole”
Source: Microbiol Spectr. 2025 Oct 21;13(12):e00730-25. doi: 10.1128/spectrum.00730-25 (PMC12671123; doi:10.1128/spectrum.00730-25)
Supplement: Figure S1 — Supplemental figure [file spectrum.00730-25-s0001.pdf]

Supplemental Figure 1

A

Compound	Structure
Collismycin 21	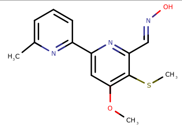
Collismycin 22	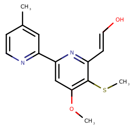
Collismycin 22-ACID	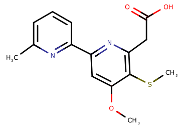
Collismycin A	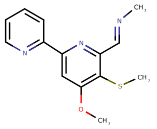
Collismycin DC	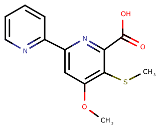
Collismycin DH	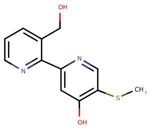
Collismycin H	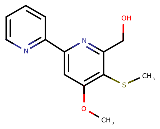
Collismycin H-BUT	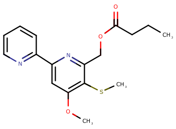
Collismycin HA	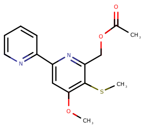
Collismycin SN	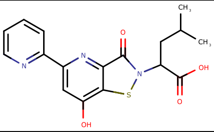
Collismycin SC	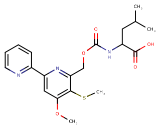
NR-4492C	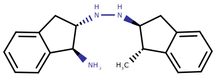
NR-4493C	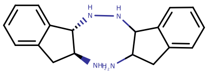

Compound	Structure
NR-5012	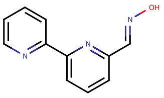
NR-6226A	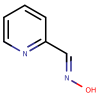
NR-6226B	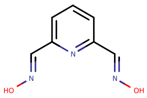
NR-6226C	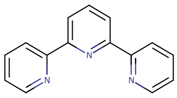
NR-6226D	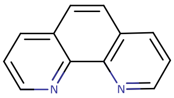
NR-6226K	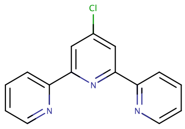
NR-6226V	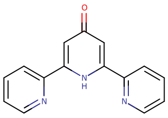
NR-6266A	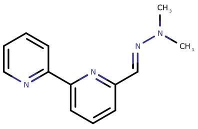
NR-6266B	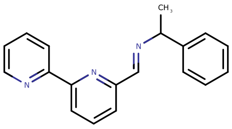
NR-6265P	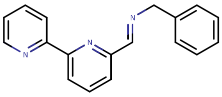
NR-6268A	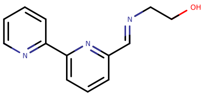
NR-6269A	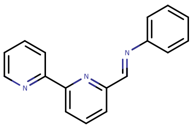
NR-6269B	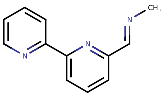
